# Lieut. Harold F. Mickley

**Published:** 1918-05

**Authors:** 


**Lieut. Harold F. Mickley,** N. Y. Homeopathic Med. College 1912, died March 16 at the Base Hospital, Camp Dix, Wrightstown, N. J. His home was in Seneca Falls.

